# On the Analysis of Fingertip Photoplethysmogram Signals

**DOI:** 10.2174/157340312801215782

**Published:** 2012-02

**Authors:** Mohamed Elgendi

**Affiliations:** School of Engineering and Information Technology, Charles Darwin University, Australia; School of Electrical and Electronic Engineering, Nanyang Technological University, Singapore; Institute of Media Innovation, Nanyang Technological University, Singapore; Affiliated with Royal Darwin Hospital, Darwin, Australia

**Keywords:** Photoplethysmography, acceleration plethysmogram, second derivative plethysmogram, digital volume pulse, ageing, artery, autonomic function, blood pressure, cardiovascular, heart rate, pulse wave analysis, vascular disease.

## Abstract

Photoplethysmography (PPG) is used to estimate the skin blood flow using infrared light. Researchers from different domains of science have become increasingly interested in PPG because of its advantages as non-invasive, inexpensive, and convenient diagnostic tool. Traditionally, it measures the oxygen saturation, blood pressure, cardiac output, and for assessing autonomic functions. Moreover, PPG is a promising technique for early screening of various atherosclerotic pathologies and could be helpful for regular GP-assessment but a full understanding of the diagnostic value of the different features is still lacking. Recent studies emphasise the potential information embedded in the PPG waveform signal and it deserves further attention for its possible applications beyond pulse oximetry and heart-rate calculation. Therefore, this overview discusses different types of artifact added to PPG signal, characteristic features of PPG waveform, and existing indexes to evaluate for diagnoses.

## INTRODUCTION

The word plethysmograph is a combination of two ancient Greek words ‘plethysmos’ which means increase [1, 2] and ‘graph’ which is the word for write [2], and is an instrument mainly used to determine and register the variations in blood volume or blood flow in the body which occur with each heartbeat.

Various types of plethysmograph exist, and each of them measures the changes in blood volume in a different manner with a specific transducer and has certain applications [3]. As shown in Table **I**, the general plethysmograph types are: water [4-8], air, strain gauge, impedance, and photoelectric.

Photoelectric plethysmography, also known as photoplethysmography and its acronym in some literature, is (PTG/PPG) and when it is called digital volume pulse, the acronym is (DVP). In this paper, the abbreviation PPG is going to be used.

PPG is easy to set up, convenient, simple and economically efficient compared to the other types of plethysmograph mentioned in Table **I** [3]. Moreover, it does not need direct contact with the skin surface, as the other plethysmograph methods.

It uses a probe which contains a light source and a detector to detect cardio-vascular pulse wave that propagates through the body.

The PPG signal reflects the blood movement in the vessel, which goes from the heart to the fingertips and toes through the blood vessels in a wave-like motion [32], as shown in Fig. (**1a**). It is an optical measurement technique that uses an invisible infrared light sent into the tissue and the amount of the backscattered light corresponds with the variation of the blood volume [2]. Hertzman was the first to find a relationship between the intensity of backscattered light and blood volume in 1938 [33]. The low-cost and simplicity of this optical based technology could offer significant benefits to healthcare (e.g. in primary care where non-invasive, accurate and simple-to-use diagnostic techniques are desirable). Further development of PPG could place this methodology among other tools used in the management of vascular disease. 

As shown in Fig. (**1a**), the wave contour of PPG signal is simple and has not been analyzed and investigated because of the difficulty in detecting changes in the phase of the inflections. Therefore , Ozawa [34] introduced the first and the second derivative of the PPG signal, as shown in Fig. (**1b**) and Fig. (**1c**) respectively, to facilitate the interpretation of the original PPG waves. The first and second derivatives of the PPG signal were developed as methods which allow more accurate recognition of the inflection points and easier interpretation of the original PPG wave.

The fingertip PPG signal reflects the blood movement in the vessel, which goes from the centre (heart) to the end (fingertips) in a wave-like motion as shown in Fig. (**1a**).

It is affected by the heartbeat, the haemodynamics and the physiological condition caused by the change in the properties of an arteriole. The effects can be observed as distortions in the wave profiles. 

Recently, analysing the PPG waveform has attracted increasing interest especially in circulatory [35] and respiratory [36] monitoring.

**Fig. (2):**
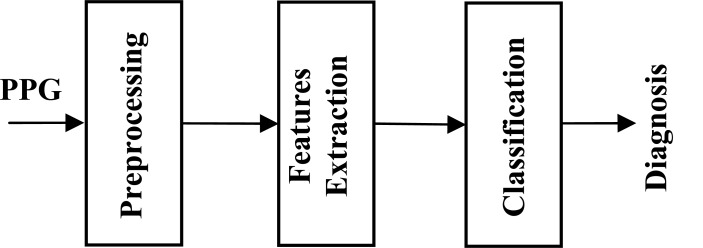
Common structure for PPG diagnostic system consists of three or four stages: 1) Preprocessing stage to emphasize the desired waves. 2) Feature extraction stage to detect the desired waves. 3) Calculate an index or a measure using the extracted features for classification and diagnosis.

**Fig. (2).** Common structure for PPG diagnostic system consists of three or four stages: 1) Preprocessing stage to emphasize the desired waves. 2) Feature extraction stage to detect the desired waves. 3) Calculate an index or a measure using the extracted features for classification and diagnosis.

As shown in Fig. (**2**), any PPG diagnostic structure consists of three stages: Pre-processing, features extraction, diagnosis/ classification. 

In this paper we discuss features and artifacts in PPG signals, and role of PPG as a diagnostic tool. 

## I PRE-PROCESSING IN PPG SIGNALS

The quality of the PPG signal depends on the location and the properties of the subject's skin at measurement, including the individual skin structure, the blood oxygen saturation, blood flow rate, skin temperatures and the measuring environment. 

These factors generate several types of additive artifact which may be contained within the PPG signals. This may affect the extraction of features and hence the overall diagnosis, especially, when the PPG signal and its derivatives will be assessed in an algorithmic fashion. The main challenges in processing the PPG signals are described as follows:

### 1) Powerline Interference

This artifact could be due to the instrumentation amplifiers, the recording system picking up ambient electromagnetic signals and other artifact.

Moreover, high frequency artifact caused by mains power sources interference is induced onto the PPG recording probe or cable. This artifact introduces a sinusoidal component into the recording. In Australia this component is at a frequency of 50Hz. 

The periodic interference is clearly displayed as a spike in Fig. (**3**) at not only its fundamental frequency of 50 Hz, but also as spikes at 100 Hz and its higher harmonics. 

### 2) Motion Artifact

This artifact is may be caused by poor contact to the fingertip photo sensor. Variations in temperature and bias in the instrumentation amplifiers can sometimes cause baseline drift as well. 

In our measurements, the body movement was limited due to the short time of measurement (20 seconds) and the fixed position of the arm during the fingertip PPG signal collection. It is hard to arrange a procedure to measure PPG signal without low frequency artifact, Fig. (**4**) shows a noisy PPG signal with poweline and motion artifacts. The low frequency artifact can be removed using a high pass filter or vice versa.

Usually the cause of motion artifacts is assumed to be due to vibrations or movement of the subject. The shape of the baseline disturbance caused by motion artifacts can be assumed to be a biphasic signal resembling one cycle of a sine wave, as shown in Fig. (**5**).

### 3) Low Amplitude PPG Signal

In general, the PPG waveform is subject to sudden amplitude changes due to the automatic gain controller which adjusts the gain of the amplifier automatically based on the amplitude of the input signal. This may cause amplitude saturation in the amplitude of the PPG waveform at a maximum or minimum value, or to rest at some random fixed value. A low amplitude PPG signal caused by the automatic gain controller is shown in Fig. (**6**). However, the reduction of PPG amplitude can be directly attributable either to a loss of central blood pressure or to constriction of the arterioles perfusing the skin

### 4) Premature Ventricular Contraction

The premature ventricular beats (PVCs) interrupt the normal heart rhythm and cause an irregular beat, as shown in Fig. (**7**). This is often felt as a "missed beat" or a "flip-flop" in the chest. PVCs are often harmless, but when they occur very often or repetitively, they can lead to more serious rhythm disturbances. This type of arrhythmia will affect the main events detection accuracy in PPG signals. Two arrows in Fig. (**7**) refer to PVC. Sometimes all of the challenges discussed could exist at the same time within the PPG signal as shown in Fig. (**8**). 

The PPG signal is complex and sensitive to artifacts. It maybe for these reasons the PPG signal has not been widely investigated beyond its use in oximetry [37]. 

## II PPG FEATURES AND ITS APPLICATIONS

The photoplethysmogram probe consist of an infrared light source (typically a photodiode emitting light at a wavelength of around 900 nm) and a photodetector (phototransistor) [38]. The light source to illuminate the tissue (e.g. skin), and a photodetector to measure the small variations in light intensity associated with changes in the blood vessels volume. The increase in blood volume indicates decrease in light intensity and vice versa [35]. 

Although the morphology of the PPG signal looks similar to the arterial pressure pulse, the wave contour is not the same. The relationship between the PPG signal and the pressure pulse has been quantified by Millasseau *et al*. [39]. 

The fingertip vasculature (blood vessels) contains an abundance of alpha adrenergic receptors which affects the arteries and veins vasoconstriction (narrowing the blood vessels).

Therefore, the peripheral blood flow will be influenced by sympathetic activity as well as temperature variations [40, 41]. This can produce significant errors, such as emphasizing local effects when relating PPG waveform features to central large artery properties. Penaz [42] developed a technique that can overcome these PPG problems including the fingertip cuff occlusions called volume clamping. His technique is used for calibrated and continuous non-invasive measurement of arterial pressure (e.g. using the Finapres device [43]).

Recently, the desire for a simple, economical, convenient, and noninvasive cardiovascular assessment techniques are the major attractive features to re-investigate the PPG [44]. Moreover, the fast development in semiconductor technology has made the PPG probe design even more attractive in terms of size, sensitivity, reliability and reproducibility. This will significantly increase the demand to apply the PPG to a large scale of human health and well-being studies. Consequently, a progress in the PPG signal processing and pulse wave analysis is expected.

Therefore, this paper will review the PPG signal processing challenges, features and the existing applications.

Features of the first and second derivative of the PPG will also be discussed. The first and second derivatives of the PPG were developed as a method to accurately recognize the critical points of the PPG. 

### A Photoplethysmogram 

The appearance of the PPG pulse is commonly divided into two phases: the anacrotic phase is the rising edge of the pulse, whereas the catacrotic phase is the falling edge of the pulse as shown in Fig. (**9**). The first phase is primarily concerned with systole, and the second phase with diastole and wave reflections from the periphery. A dicrotic notch, shown in Fig. (**9**), is usually seen in the catacrotic phase of subjects with healthy compliant arteries. A number of features based on the PPG have been described in literature.

#### 1) Systolic Amplitude:

As shown Fig. (**9**), the systolic amplitude (x) is an indicator of the pulsatile changes in blood volume caused by arterial blood flow around the measurement site [45, 46]. Systolic amplitude has been related to stroke volume [47]. Dorlas and Nijboer found that systolic amplitude is directly proportional to local vascular distensibility over a remarkably wide range of cardiac output [48]. It is also has been suggested that systolic amplitude is potentially a more suitable measure than pulse arrival time for estimating continuous blood pressure [49].

Table **II** summarises the several physiological and pharmacological factors, which influence the systolic amplitude in PPG signals. 

#### 2) Pulse Width:

the pulse width in the PPG wave is shown in Fig. (**9**). Awad *et al.* [50] used the pulse width as the pulse width at the half height of the systolic peak. They have suggested that the pulse width correlates with the systemic vascular resistance better than the Systolic amplitude.

#### 3) Pulse Area:

the pulse area is measured as the total area under the PPG curve. Seitsonen *et al.* [51] found the PPG area response to skin incision to differ between movers and non-movers.

Wang *et al.* [52] have divided the pulse area into two areas at the dicrotic notch. They found that the ratio of the two areas, see Fig. (**10**), can be used as an indicator of total peripheral resistance. This ratio is called the inflection point area ratio ( *IPA*) and is defined as

(1) $$\mathrm{IPA}=\frac{\mathrm{A2}}{\mathrm{A1}}$$

#### 4) Peak to Peak Interval:

The distance between two consecutive systolic peaks will be referred to as *Peak-Peak interval*, as shown in Fig. (**11**).

The R-R interval in the ECG signal correlates closely with the *Peak-Peak interval* APG signal as both represent a completed heart cycle. The *Peak-Peak interval* has been used to detect the heart in PPG signals [53-56].

#### 5) Pulse Interval:

The distance between the beginning and the end of the PPG waveform, as shown in Fig. (**11**). The *Pulse interval* is usually used instead of the *Pulse interval* when the diastolic peaks are more clear and easier to detect compared to the systolic peak. 

Poon *et al*. [57] suggested that ratio of *Pulse interval *to its systolic amplitude could provide an understanding of the properties of a person's cardiovascular system. In 2008, Lu *et al. *[58] compared the HRV using the *Pulse interval* in PPG signals with the HRV using R-R intervals in ECG signals. Their results demonstrated that HRV in PPG and ECG signals are highly correlated. They strongly suggested that PPG signals could be used as an alternative measurement of HRV.

#### 6) Augmentation Index:

The augmentation pressure (AG) is the measure of the contribution that the wave reflection makes to the systolic arterial pressure, and it is obtained by measuring the reflected wave coming from the periphery to the centre. Reduced compliance of the elastic arteries causes an earlier return of the ‘reflected wave’, which arrives in systole rather than in diastole, causing a disproportionate rise in systolic pressure and an increase in pulse pressure, with a consequent increase in left ventricular after load and a decrease in diastolic blood pressure and impaired coronary perfusion.

Takazawa *et al*. [59] defined the augmentation index ( *AI* ) as the ratio of y to x as follows:

(2) $$\mathrm{AI}=\frac{y}{x}$$

As shown Fig. (**9**), y is the height of the late systolic peak and x is the early systolic peak in the pulse. 

Padilla *et al* [60] used the *RI* as a reflection index as follows:

(3) $$\mathrm{RI}=\frac{y}{x}$$

Rubins *et al.* [61] used the reflection index as in equation 3 and introduced an alternative augmentation index as follows

(4) $$\mathrm{AI}=\frac{x-y}{x}$$

#### 7) Large Artery Stiffness Index:

The systolic component of the waveform arises mainly from a forward-going pressure wave transmitted along a direct path from the left ventricle to the finger. The diastolic component arises mainly from pressure waves transmitted along the aorta to small arteries in the lower body, from where they are then reflected back along the aorta as a reflected wave which then travels to the finger. The upper limb provides a common conduit for both the directly transmitted pressure wave and the reflected wave and, therefore, has little influence on their relative timing. As shown in Fig. (**12**), the time delay between the systolic and diastolic peaks (or, in the absence of a second peak, the point of inflection) is related to the transit time of pressure waves from the root of the subclavian artery to the apparent site of reflection and back to the subclavian artery. This path length can be assumed to be proportional to subject height (*h*). 

Therefore, Millasseau *et al* [62] formulated an index of the contour of the PPG ( *SI* ) that relates to large artery stiffness.

(5) $$\mathrm{SI}=\frac{h}{\Delta T}$$

They have examined the timing of discrete components of the PPG to formulate an index of the contour of the PPG expected to relate to large artery stiffness *SI*. As shown in Fig. (**13**), the time delay between the systolic and diastolic peaks decreases with age as a consequence of increased large artery stiffness and increased pulse wave velocity of pressure waves in the aorta and large arteries. Therefore, Millasseau *et al.* [62] proved that the *SI* increases with age.

In order to facilitate the interpretation of the original PPG waves, Ozawa differentiated the PPG signals to be able to analyse the PPG wave contour [59]. 

### B First Derivative Photoplethysmogram 

The first derivative is hardly used in literature and its main features are:

#### 1) Diastolic point definition:

Millasseau *et al* [62] defined the diastolic point as the point at which the first derivative of the waveform is closest to zero, shown in Fig. (**14**). 

#### 2) ΔT calculation:

ΔT is the peak-to-peak time which is related to the time taken for the pressure wave to propagate from the heart to periphery and back. The time between the systolic and diastolic peaks is ΔT. The definition of ΔT depends on the PPG waveform as its contour varies with subjects. When there is a second peak as in Fig. (**14a**), ΔT is defined as time between the two maxima. 

In other words, ΔT is the time between the two positive to negative zero-crossings of the derivative as in Fig. (**14b**). However, in some PPG waveforms, there is no clear second peak. In this case, ΔT is defined as the time between the peak of the waveform and the inflection point on the down slope of the waveform which is a local maximum of the first derivative. 

#### 3) Crest time (CT) calculation:

Crest time is the time from the foot of the PPG waveform to its peak; see Fig. (**14b**).

Alty *et al* [63] proved that the crest time is a useful feature for cardiovascular disease classification. They developed a method to classify subjects into high and low pulse wave velocity (equivalent to high and low cardio vascular disease risk) using features extracted from the PPG. They found that peak-to-peak time (ΔT), crest time (CT), and stiffness index ( *SI* =*h*/ ΔT) were the best features for accurate classification of cardiovascular disease using the first derivative of the PPG. They used sets of these features for classification. Using a support vector machine based classifier they achieve a classification result of 87.5%. 

### C Second Derivative Photoplethysmogram

The second derivative is more commonly used than the first derivative. In literature, the second derivative of photoplethysmogram is also called the acceleration plethysmogram because it is an indicator of the acceleration of the blood in the finger. Three abbreviations are commonly used for the second derivative: SDPTG (Second Derivative of the PhothophleThysmoGram), SDDVP (Second Derivative of Digital Volume Pulse) and APG (Acceleration PlethysmoGram). 

As shown in Fig. (**15**), The waveform of the APG includes four systolic waves and one diastolic wave, namely ***a***-wave (early systolic positive wave), ***b***-wave (early systolic negative wave), ***c***-wave (late systolic reincreasing wave), ***d***-wave (late systolic redecreasing wave) and ***e***-wave (early diastolic positive wave). The ***e***-wave represents the dicrotic notch as shown in Fig. (**15**). Its location corresponds to the closure of the aortic valve and subsequent retrograde blood flow, and can be used to monitor cardiac function [64, 65].

In literature, the second derivative of photoplethysmogram (SDPTG) has been called acceleration plethysmogram (APG) or second derivative of digital volume pulse (SDDVP). In this paper, the abbreviation APG is going to be used.

The height of each wave was measured from the baseline, with the values above the baseline being positive and those under it negative. The ratios of the height of the each wave to that of the *a*-wave (*b/a*, *c/a*, *d/a* and *e/a*) are usually used for wave analyses [66].

The second derivative of the finger PPG waveform is used to stabilize the baseline and enable the individual features to be visualized and detected easily. The APG main features are:

#### • Ratio b/a

Takazawa *et al*. [59] demonstrated that the *b/a *ratio reflects increased arterial stiffness, hence the *b*/*a *ratio increases with age. Imanaga *et al*. [67] provided a direct evidence that magnitude of *b/a* of the APG is related to the distensibility of the peripheral artery, and suggest that the magnitude of *b/a* is a useful non-invasive index of atherosclerosis and altered arterial distensibility. 

Aiba *et al*. [68] suggested the parameter *-b/a* in the exposure group dose dependently decreased with increases in length of working career (duration of exposure to lead) and blood lead concentration (Pb-B). The parameter *-b/a* significantly decreased in subjects with working careers of 5 years or more and in subjects whose Pb-B was 40µg/100 ml or more. While Šimek *et al. *[69] found that the *b/a* index discriminates independently between subjects with essential hypertension and healthy controls. 

Otsuka *et al.* [70] found that the *b/a*, is positively correlated to the Framingham risk score. Framingham risk score has been used to estimate individual risk of cardiovascular heart disease. Their results suggest that *b/a* index might contribute to the discrimination of the high-risk subjects for cardiovascular heart disease 

Baek *et al* [71] confirmed that the *b/a* ratio increases with age.

#### • Ratio c/a

Takazawa *et al*. [59] demonstrated that the *c/a *ratio reflects decreased arterial stiffness, hence the *c*/*a *ratio decreases with age. The *c/a* index was also used by Šimek *et al *(2005) [69] who found that the *c/a* index distinguishes subjects with essential hypertension from healthy controls. Baek *et al* [71] found that the *c/a* ratio decreases with age just as the *b/a *ratio, described above. 

#### • Ratio d/a

In 1998, Takazawa *et al*. [59] demonstrated that the *d/a *ratio reflects decreased arterial stiffness, hence the *d*/*a *ratio decreases with age. Moreover, they found the *-d/a* ratio is a useful index for the evaluation of vasoactive agents, as well as an index of left ventricular afterload. Baek *et al* [71] confirmed that the *d/a* ratios decreases with age. 

#### • Ratio e/a

Takazawa *et al*. [59] demonstrated that an increase of the *e/a *ratio reflects decreased arterial stiffness, and that the *e*/*a *ratio decreases with age. Baek *et al* [71] confirmed that the *e/a* ratios decreases with age. 

#### • Ratio (b-c-d-e)/a

Takazawa *et al*. [59] found that the *(b - c - d - e)/a* index, increases with age and may be useful for evaluation of vascular aging and for screening of arteriosclerotic disease. Kimura *et al*. [72] calculated the vascular age as 45.5*(b - c - d - e)/a* + 65.9 years old.

#### • Ratio (b-e)/a

Baek *et al* [71] suggested the *(b - e)/a* ratio as the APG aging index instead of *(b - c - d - e)/a*, when the *c* and *d* waves are missing.

#### • Ratio (b-c-d)/a

Ushiroyama *et al. *[74] reported that patients with a sensation of coldness showed an improvement of the APG index *(b - c - d)/a* upon treatment with a herbal supplement.

#### • Ratio (c+d-b)/a

Sano *et al*. [75] proposed a more comprehensive aging index *(c + d - b)/a* . It increases with age.

Sano *et al.* distinguished seven main categories of APG signals depending on the waveforms as shown in Fig. (**16**).

#### • a-a interval

The R-R interval in the ECG signal correlates closely with the *a-a* interval in APG signal as both represent a completed heart cycle. In 2007, Taniguchi* et al. *[76] used the *a-a* interval instead of the R-R interval to evaluate the surgeon's stress. In 2010, Elgendi *et al. *calculated the heart rate and heart rate variability from the APG signals [77-80].

#### • APG waveform

The shape of the APG waveform has been categorized into seven types, A to G as shown in Fig. (**16**). The shape of the APG waveform can be described as in Table **III**. Type A is often observed in healthy young people indicating good circulation. While, Type D-G is often observed in patients with cerebrovascular disease, ischemic heart disease, breast tumour and uterine diseases. The changes from D to G reflect the disease development.

Nousou* et al. *[69] developed a diagnostic system using APG and Self-Organizing Maps (SOM). They needed to adjust the original APG signal in order to be classified correctly by the SOM. The *b *and the *d *wave had to be shifted along the time axis. They used a similar classification as Sano *et al.* [75] as shown in Fig. (**16**).

#### • Section of the APG waveform

Tokutaka* et al*. [81] also developed a diagnostic tool to describe the general state of health. They used the first section of the APG signal after the *a *peak in combination with self organising maps. Their approach was similar to Nousou* et al.*[69].

#### • Chaos Attractor

Iokibe* et al*. [82] used the APG of healthy subjects and of patients with a different diseases, varying from a common cold to pneumonia, intracerebral hemorrhage and acute poisoning. Their aim was to find an indicator for the seriousness of the disease, the disease state. They applied chaos theory to the APG signals.

Fujimoto* et al. *[83] proposed a criterion which combines two evaluations based on chaos theory; the trajectory parallel measure method and the size of neighbourhood space in chaos attractor to diagnose stress using the APG. 

## III DISCUSSION

Photoplethysmography has the advantage of being a low cost, simple and portable technology which can be used in primary health care and remote clinics. This review has described a number of features of the photoplethysmogram and their potential applications. Two indices based on the original photoplethysmogram (PPG) signal have been described: the augmentation index and large artery stiffness index. Features based on the first derivative of the photoplethysmogram are: the diastolic point, the peak to peak time (ΔT), and the crest time. The first derivative of the PPG can also be used to calculate the augmentation index and the large artery stiffness index more accurately.

Most indices are based on the second derivative of the finger photoplethysmogram (APG) which seems to provide more information than the first derivative of PPG. The indices calculated from the APG waveforms are reported to correlate closely with the distensibility of the carotid artery [67], age [59], the blood pressure [84], the estimated risk of coronary heart disease [70], and the presence of atherosclerotic disorders [85]. Some of the photoplethysmogram indices have been calculated with different formulas. For example the aging index can be calculated as *(b - c - d - e)/a, (b - e)/a* or *(c + d - b)/a .*. A number of indices are reported to indicate vascular stiffness; the *b/a *index increases with increasing arterial stiffness while the* c/a, d/a *and *e/a *indices decrease. At this stage it is unclear which of these indices is most informative. Sometimes the same feature is used as a measure of different but potentially related physiological variables. The *b/a *ratio has been used as an indicator of arterial stiffness, hypertension, vascular aging and risk of cardiovascular disease. 

Most research relating to the APG has been done in Japan. In addition to cardiovascular risk factors, the APG has also been described as a potential diagnostic tool for other disorders, varying from a sensation of coldness [74] and stress experienced by surgeons [76] to exposure to lead [70], pneumonia, intracerebral haemorrhage and acute poisoning [82]. This has its origins in Eastern medicine where the pulse is considered a very important diagnostic variable. Self organising maps and chaos theory have been applied to find a measure of the disease state or the general state of health [69], [81], [82].

Currently a full understanding of the diagnostic value of the different features of the PPG signal is still lacking and more research is needed. 

## IV CONCLUSION

This review discussed the photoplethysmography technology and demonstrated their potential diagnostic applications.

A common structure of any PPG diagnostic system consists of three stages preprocessing, feature extraction, and diagnosis. The main focus of this review was the preprocessing and feature extraction stages.

In the preprocessing stage, different artifact sources affecting the PPG signal are described. The sources of artifact can be the power line interface, motion artifacts, low amplitude, and the existence of arrhythmia.

In the feature extraction stage, the characteristics of the PPG waveform and its derivatives have been clarified. Features of the PPG signal have been discussed. These features may be calculated based on the original signal or on the first or second derivative of the PPG signal. Taking the first and second derivatives of the PPG signals may help in detecting the informative inflection points more accurately. Different features have been used as indicators for the same physiological variables. Several vascular stiffness and aging indices have been described and it is currently not clear which of these is most informative. Some features have been used as indicators of different but potentially related cardiovascular variables. Features of the second derivative of the PPG have also been described in literature as indicators for the general state of health. Moreover, the paper presented the most common PPG indexes in the clinical assessment. There is no doubt that these indexes have the potential to be applied to many other pathological studies.

Photoplethysmography is a promising technology due to its simplicity, low cost and non-invasiveness. It has potential for early screening for various atherosclerotic pathologies and could be useful for regular GP-assessment or even self-monitoring. However, a full understanding of the diagnostic value of the different features is still lacking and more research is needed.

## Figures and Tables

**Fig. (1):**
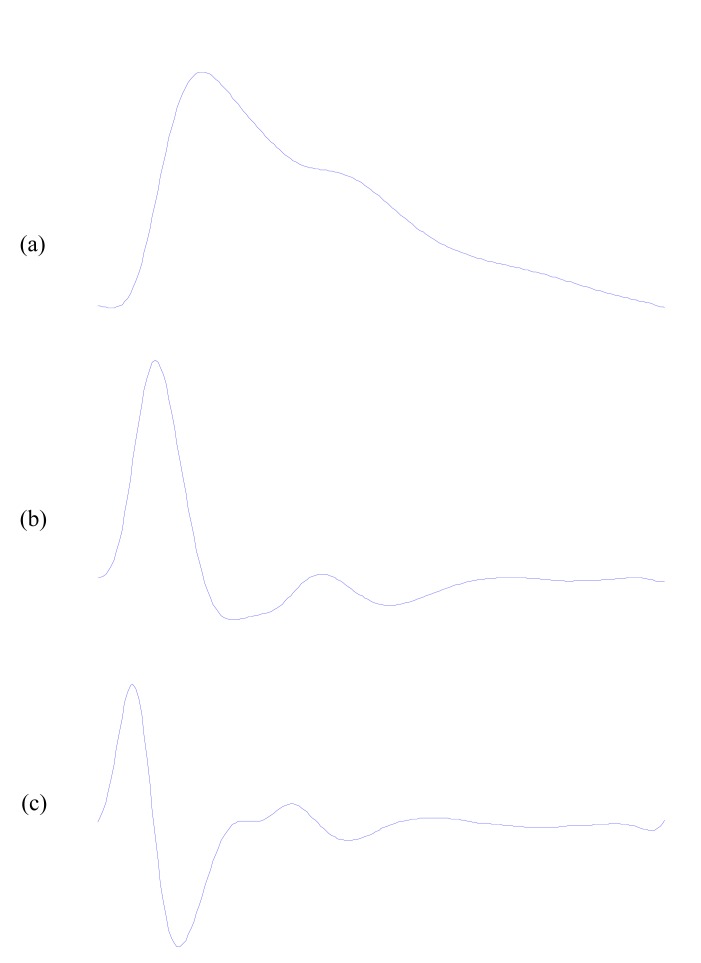
Signal Measurements (**a**) Original fingertip photoplethysmogram (**b**) first derivative wave of photoplethysmogram (**c**) second derivative wave of photoplethysmogram.

**Fig. (3):**
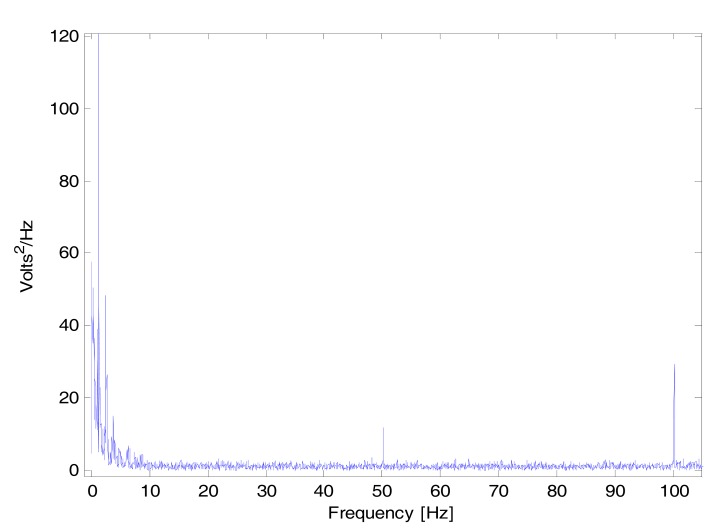
Power Spectrum of the PPG signal. The spectrum illustrates peaks at the fundamental frequency of 50 Hz as well as the second harmonic at 100 Hz.

**Fig. (4):**
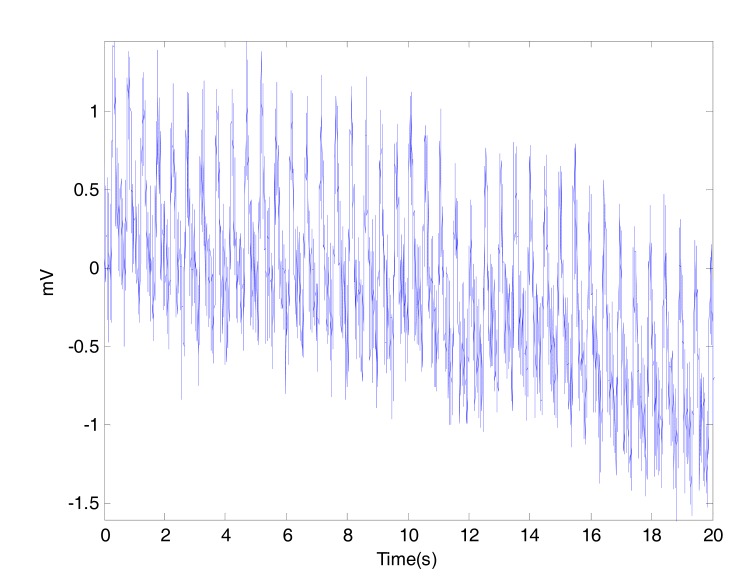
Powerline and motion artifacts in PPG.

**Fig. (5):**
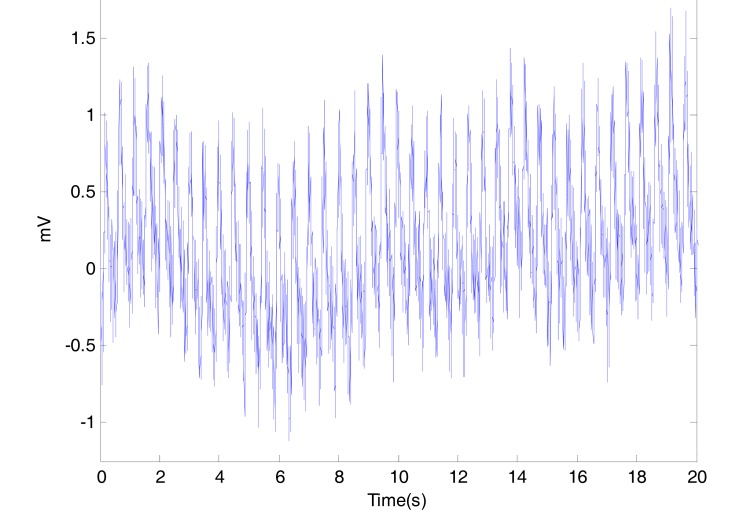
Baseline wandering in PPG.

**Fig. (6):**
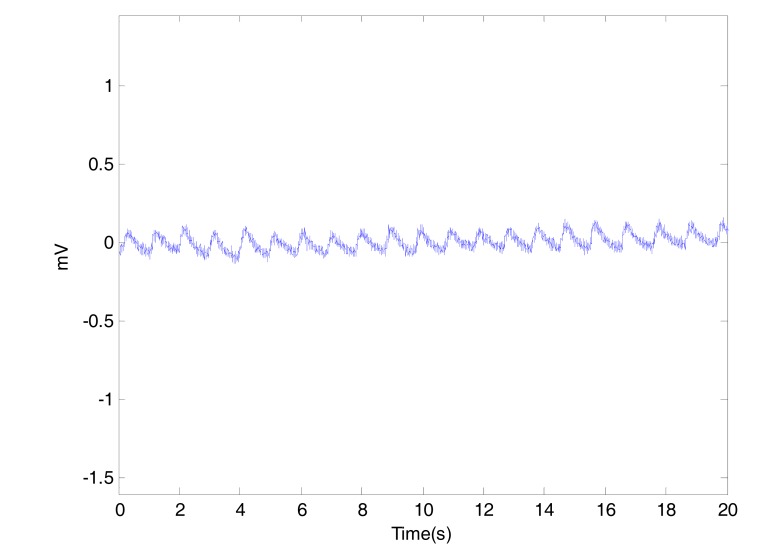
Low Amplitude PPG signals. Generally, the low amplitude PPG signal is most likely related to the automatic gain controller, but it can be caused due to: bad connectivity between fingertip probe and the finger, loss of central blood pressure, or constriction of the arterioles. Detecting the heart beats in low amplitude PPG signals is considered difficult.

**Fig. (7):**
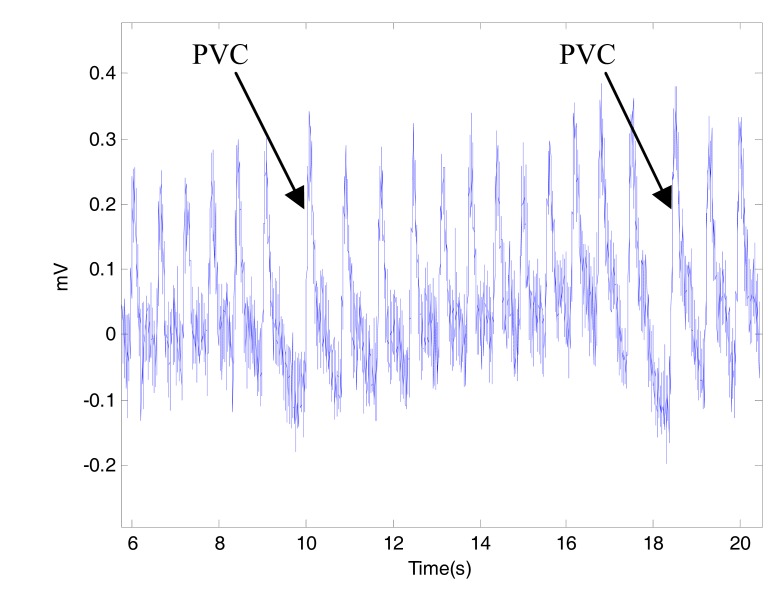
Premature Ventricular Contraction. It is clear that detection of heart beats in PPG signals will be challenging with the existence of PVCs.

**Fig. (8):**
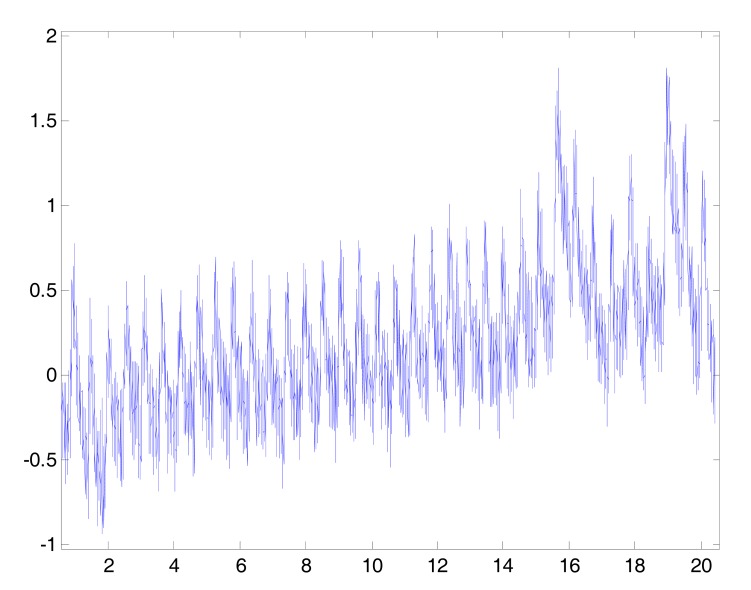
Artifacts in PPG signals. This PPG sample contains different challenges in analyzing PPG signals: motion artifacts, muscle artifact, arrhythmia, high frequency artifact, and low amplitude.

**Fig. (9):**
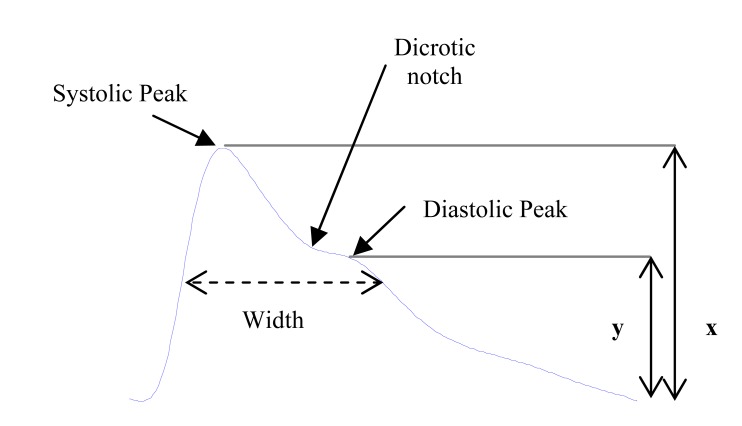
A typical waveform of the PPG and its characteristic parameters, whereas the amplitude of the systolic peaks is x while y is the amplitude of the diastolic peak.

**Fig. (10):**
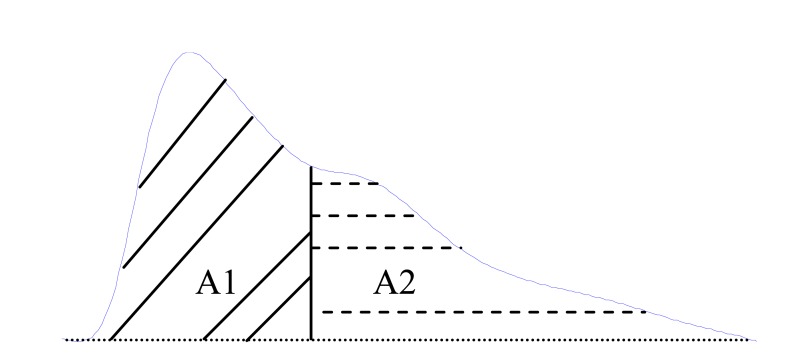
Original fingertip photoplethysmogram. A1 and A2 are the areas under the whole PPG wave separated at the point of inflection. Thus, the inflection point area ration can be calculated as the division of A2 by A1.

**Fig. (11):**
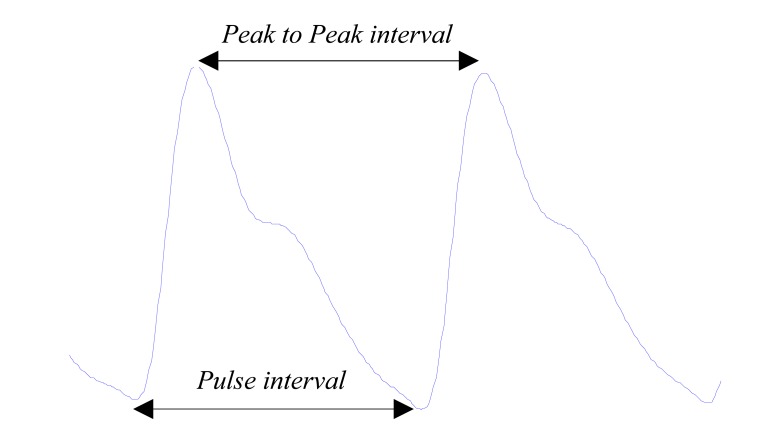
Two consecutive PPG waves.

**Fig. (12):**
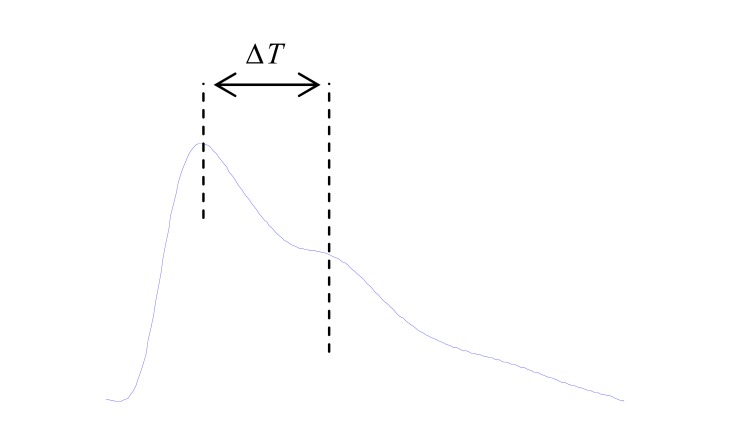
Typical waveform of the PPG and its *ΔT* feature.

**Fig. (13):**
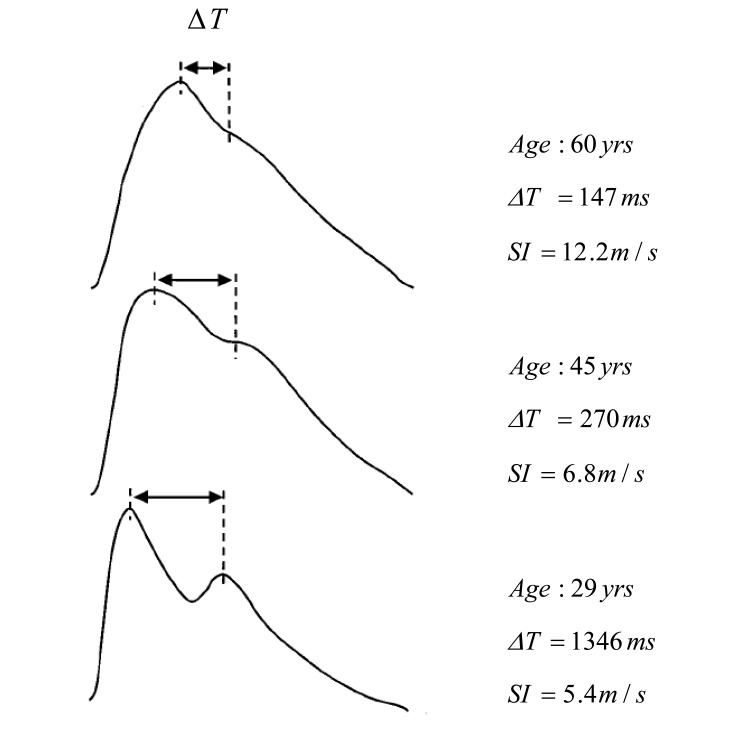
Typical PPG waveforms show the parameters changes with age [62].

**Fig. (14):**
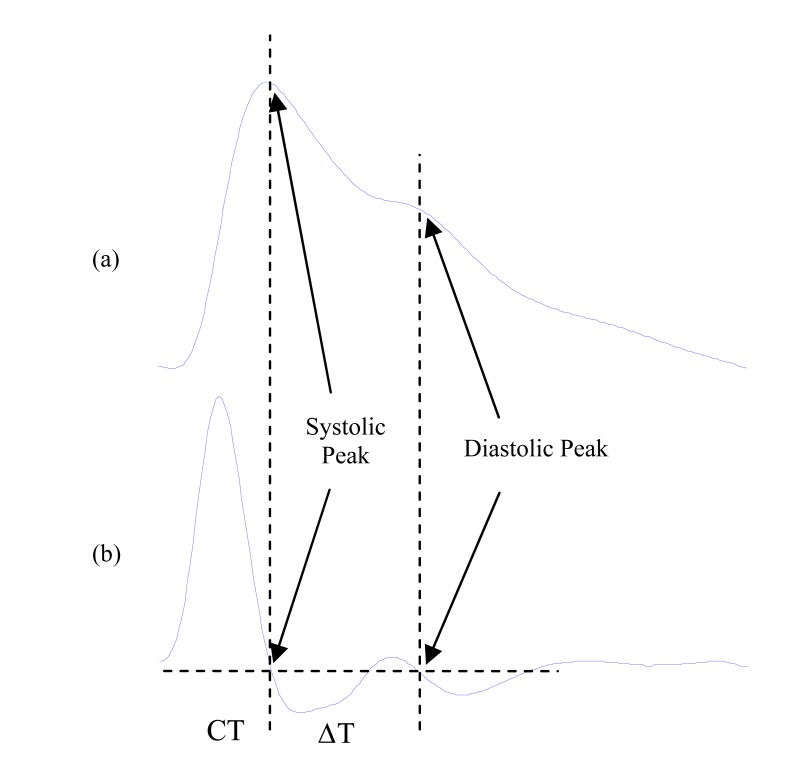
Signal Measurements (**a**) Original fingertip photoplethysmogram (**b**) first derivative wave of photoplethysmogram

**Fig. (15):**
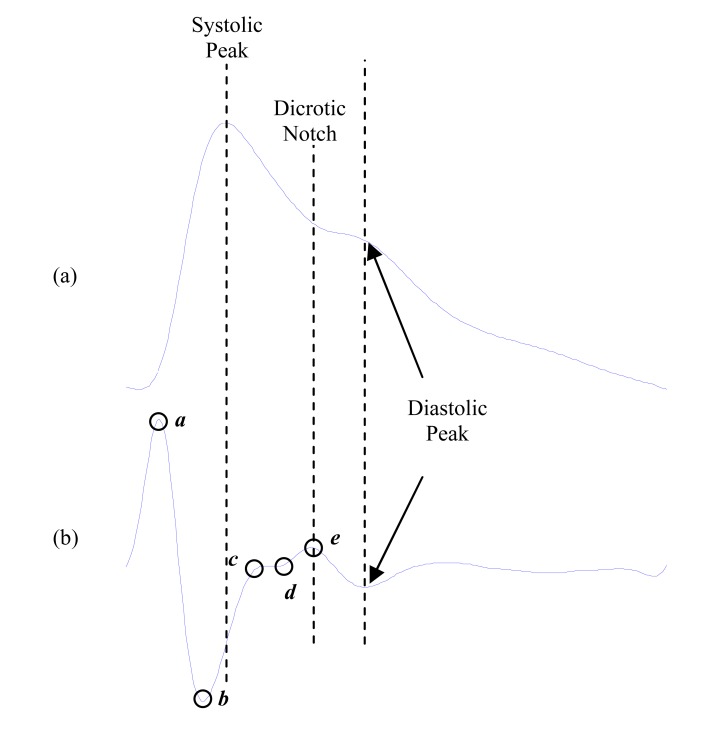
Signal Measurements (**a**) Original fingertip photoplethysmogram (**b**) second derivative wave of photoplethysmogram.

**Fig. (16):**
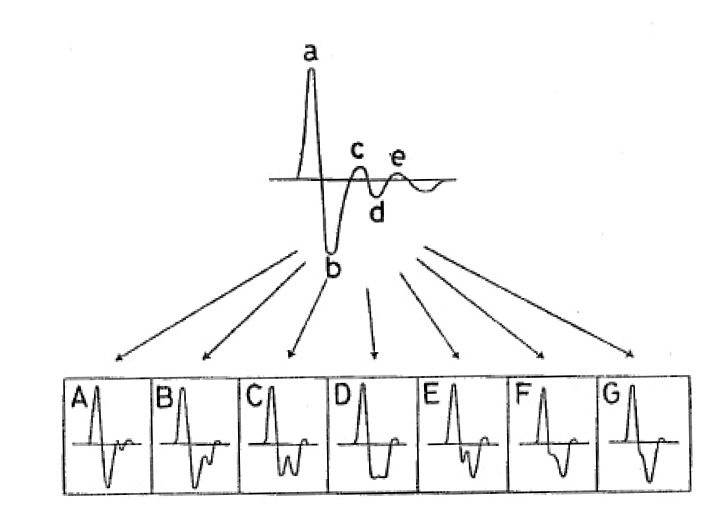
APG waveforms and types of photoplethysmogram [73]. There are different types of APG waveforms. The first APG waveform A (far left) refers to good circulation, whereas the amplitude of *b* wave is lower than *c* wave. The last APG waveform G (far right) refers to distinctively bad circulation, whereas the amplitude of *c* wave is lower than *b* wave.

**Table I.** Types of Plethysmograph and Its Application

Type	Transducer	Standard Applications
**Water**	Water-filled cuff [4, 5] Water-filled body [6, 7] Water-filled chamber [8]	Measuring penile blood flow [4, 5]. Measuring Pulmonary Capillary Blood Flow [6, 7]. Measuring maximal blood flow [8].
**Air**	Air-filled cuff	Evaluation of venous hemodynamics [9, 10]. Measures parameters of global venous function [9] like: calf venous volume venous filling index ejection fraction residual volume fraction
**Strain Gauge**	Fine rubber tube (filled with mercury)	Assessment of capillary filtration [11]. Assessment of volume changes in venous diseases [11]. Identifying limbs with suspected venous incompetence [12]. Evaluation of peripheral circulation in spinal cord injury cases [12]. Evaluation of acute and chronic venous insufficiency [13]. Evaluation of peripheral vascular disease [14]. Measurement of deep venous thromboses [15, 16].
**Impedance **	Electrodes	Detection of blood flow disorders [17, 18]. Assessment of fat-free mass of the human body [19].
**Photoelectric**	Photo detectors	Monitoring of heart and respiratory rates [20]. Monitoring of oxygen saturation [21, 22]. Assessment of blood vessel viscosity [23]. Assessment of venous function [24]. Measuring the ankle pressure [25]. Measuring genital responses [26] Assessment of venous reflux [27]. Measuring cold sensitivity [28, 29]. Measuring blood pressure [30]. Assessment of cardiac output [31].

**Table II.** Different Factors Affecting the Systolic Amplitude [37]

Systolic Peak Amplitude	Factor	Effect
**Low**	Relative elevation of measurement site	Decreased blood volume pulsations and decreased venous blood volume
	Arterial blood pressure increase due to increased peripheral resistance	Decreased blood volume pulsations
	Severe hypovolaemia	Decreased blood volume pulsations
	(Local) hypothermia	Peripheral vasoconstriction
	Sympathetic activation (e.g. stress, cold)	Peripheral vasoconstriction
	Vasoconstrictors (e.g. Noradrenaline)	Peripheral vasoconstriction
**High**	Arterial blood pressure increase due to increased cardiac output	Increased blood volume pulsations
	Most anaesthetics	Peripheral vasodilatation
	Epidural anaesthesia	Peripheral vasodilatation

**Table III.** APG Wave Form Types [73]

Beat Type	Description
**A**	Good circulation
**B**	Good circulation but deteriorating
**C**	Poor circulation
**D-G**	Distinctively bad circulation
